# The angle of the tines before the pull and hold test predicts engagement of the tines in Micra leadless pacemaker implantation

**DOI:** 10.1002/joa3.12797

**Published:** 2022-11-23

**Authors:** Akira Mizukami, Shota Miyakuni, Ryo Nakada, Tetsuya Kobayashi, Takuya Kawakami, Koki Takegawa, Hirofumi Arai, Jiro Hiroki, Kenji Yoshioka, Hirofumi Otani, Maki Ono, Shu Yamashita, Daisuke Ueshima, Makoto Suzuki, Akihiko Matsumura, Masahiko Goya, Tetsuo Sasano

**Affiliations:** ^1^ Department of Cardiology Kameda Medical Center Chiba Japan; ^2^ Department of Cardiology Yokohama City Minato Red Cross Hospital Yokohama Japan; ^3^ Department of Cardiology Yokohama Minami Kyosai Hospital Yokohama Japan; ^4^ Department of Cardiovascular Medicine Tokyo Medical and Dental University Tokyo Japan

**Keywords:** angle, engagement, leadless pacemaker, Micra, tines

## Abstract

**Background:**

Micra leadless pacemaker is secured to the myocardium by engagement of at least 2/4 tines confirmed with pull and hold test. However, the pull and hold test is sometimes difficult to assess. This study was performed to evaluate whether the angle of the tines before the pull and hold test predicts engagement of the tines in Micra leadless pacemaker implantation.

**Methods:**

We retrospectively enrolled 93 consecutive patients (52.7% male, age 82.4 ± 9.4 years), who received Micra implantation from September 2017 to June 2020 at our institution. After deployment and before the pull and hold test, the angle of the visible tines to the body of the pacemaker was measured using the RAO view of the fluoroscopy image. The engagement of the tines was then confirmed with the pull and hold test.

**Results:**

A total of 326 tines were analyzed. The angle of the engaged tines was significantly lower than the non‐engaged tines (9.2 degrees [4.0–14.0] vs. 16.6 degrees [14.2–18.8], *p* < .0001). All tines with angles <10 degrees were engaged. In higher angles, engagement could not be predicted.

**Conclusion:**

A low angle of the tines before the pull and hold test can predict engagement of the tines in Micra leadless pacemaker implantation. The tines which are already open after deployment may be presumed that they are engaged.

## Graphical abstract

The angle of the tines before the pull and hold test can predict engagement of the tines in Micra leadless pacemaker implantation. The angle of the engaged tines was significantly lower than the non‐engaged tines. All tines with angles <10 degrees were engaged.

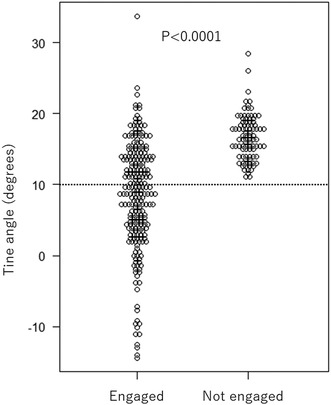

## 1 INTRODUCTION

Micra leadless pacemaker (Medtronic) is a breakthrough in technological advances of cardiac pacing. Since the leadless pacemaker itself is implanted to the heart and has no leads and the subcutaneous pocket, it has advantages over conventional pacemakers regarding complications related to the leads and the pocket. It is also free from complications related to lead insertion and vascular obstruction.^1^, ^2^, ^3^, ^4^

**FIGURE 1:**
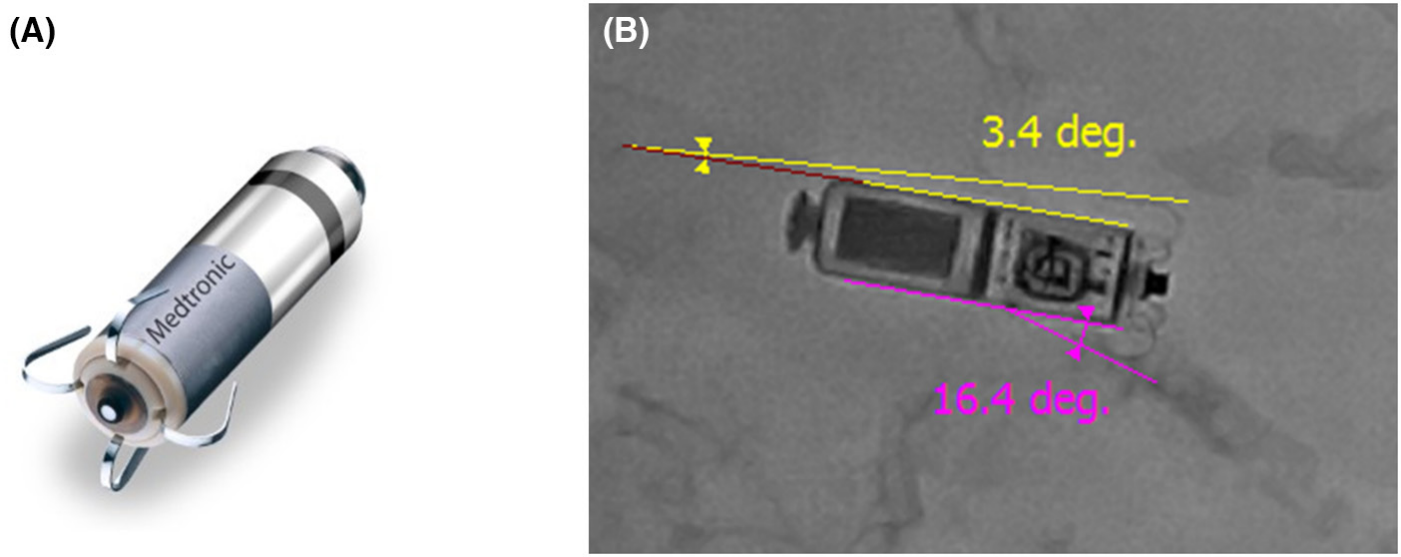
Micra leadless pacemaker and tine angles. (A) Micra leadless pacemaker and its tines. Four electrically isolated nitinol tines are placed at the tip of the device. Nitinol is a shape memory metal, and all tines are pre‐shaped to directed inward toward the body of the device. (B) Assessment of the angle of the tines with right anterior oblique (RAO) view of the cine‐image. The angle between each visible tine and the body of the pacemaker was calculated before the pull and hold test. In this representative case, the upper tine with an angle of 3.4 degrees was engaged. On the contrary, the bottom tine with an angle of 16.4 degrees was not engaged. The differences in tine angles of the opposing pair of tines were also calculated (16.4–3.4 = 13 degrees in this patient).

Lead dislodgement is the most common major complication of conventional pacemaker implantation,^5^ in which leadless pacemakers are unrelated.^1^, ^2^, ^3^, ^4^ However, the leadless pacemaker itself carries the risk of dislodgement. The Micra leadless pacemaker is secured to the myocardium by 4 electrically isolated nitinol tines placed at the tip of the device (Figure 1A).^1^, ^6^, ^7^, ^8^ Nitinol is a shape memory metal, and all tines are pre‐shaped to direct inward toward the body of the device. Engagement of at least 2/4 tines confirmed with the pull and hold test is thought to be satisfactory in minimizing the risk of device dislodgement.^6^, ^9^ Due to the shape memory of the tines, tines will open with the pulling force only if the tines are engaged in the myocardium.

The pull and hold tests are sometimes difficult to assess due to unclear images and movement of the tines. Unclear assessment of engagement may lead to device dislodgement requiring retrieval^10^, ^11^, ^12^ and unnecessary redeployments which may lead to cardiac injury and tamponade.

The engaged tines are sometimes already open before the pull and hold test, presumably due to myocardial tissue trapped between the tine and the body of Micra. This may suggest the engagement of the tines. However, studies comparing the angle of the tines before the pull and hold test and engagement of the tines are lacking.

We thought to evaluate the relationship between the angle of the tines before the pull and hold test and the engagement of the tines in patients receiving Micra leadless pacemaker implantation.

## 2 METHODS

### 2.1 Study population

This retrospective single‐center study enrolled 93 consecutive patients who underwent Micra leadless pacemaker implantation from September 2017 to June 2020 at Kameda Medical Center in Japan. The indications for pacemaker implantation, as well as the choice of leadless pacemaker, were decided according to the 2011 and 2019 Japanese Circulation Society and Japanese Heart Rhythm Society Guideline on Non‐Pharmacotherapy of Cardiac Arrhythmias.^13^, ^14^ The patient background including activity of daily living (ADL), cognitive function, ability to follow instructions, and frailty was also considered when considering a leadless pacemaker.

### 2.2 Implantation procedure

The right femoral vein was punctured with ultrasound guidance, and an 8Fr sheath was introduced. An arterial line was also routinely inserted from the right femoral artery to monitor the blood pressure during the procedure. A 0.035 mm radifocus guidewire (Terumo) was inserted into the superior vena cava, and venography from the right femoral vein was performed to assess the anatomy of the access route to the right atrium, as well as the right heart chambers. The wire was changed to the Amplatz Super Stiff wire (Boston Scientific) with the use of a multipurpose catheter. The right femoral vein was dilated with a dilator, followed by insertion of the 23Fr Micra introducer sheath (Medtronic). Through the introducer sheath, Micra leadless pacemaker was implanted to the right ventricle with the use of the delivery catheter. The right ventricular septum was always targeted for implantation. Right anterior oblique (RAO) and left anterior oblique (LAO) views were always used. After deployment, a pull and hold test was performed. Engagement of at least 2/4 tines was thought to be satisfactory. Device parameters were checked. If the parameters are unsatisfactory, recapture and redeployment were performed until satisfactory results were obtained.

### 2.3 Assessment of tine engagement and tine angle

Two or more operators were always involved in the implantation procedure, and the tine engagement was always assessed and discussed by all operators until agreement was obtained during the procedure. For this study, the independent observer who was blinded to the baseline characteristics and engagement assessment of the operator reviewed the cine‐image of the pull and hold test. Tine engagement was assessed and recorded. If the discrepancy was observed between the assessment by the observer and the operator, the second observer was introduced. If multiple deployments were performed in a single patient, all deployments were assessed individually.

The RAO cine‐image before the pull and hold test for each deployment was also assessed by an independent observer who was blinded to the baseline characteristics and the results of the pull and hold test. The angle between the body of Micra and the tines was measured by the QAngio XA software (Medis Medical Imaging Systems) (Figure 1B) and recorded.

RAO cine‐image, which could assess the body and the tines longitudinally was used for this study. LAO cine‐image was not used due to the difficulty of assessment of the angle of the tines. Tines that overlap with the body of Micra and the other tines were excluded from the analysis.

### 2.4 Data analysis

Data at the time of implantation procedure were collected, including age, sex, body mass index (BMI), height, weight, diagnosis for implantation (complete AV block, advanced AV block, sick sinus syndrome [SSS] type I /II, SSS type III, and atrial fibrillation with bradycardia), past history (hypertension, diabetes mellitus, heart failure, atrial fibrillation, ischemic heart disease, chronic obstructive pulmonary disease [COPD], and hemodialysis), and left ventricular ejection fraction (LVEF) on transthoracic echocardiography. Hypertension and diabetes mellitus were scored based on the previous diagnosis and initiation for therapy. Heart failure, atrial fibrillation (AF), ischemic heart disease, COPD, and emodialysis were scored based on the previous history.

The relationship between the tine engagement and the angle of the tines before the pull and hold test was evaluated.

Ethical approval was obtained from the Kameda Medical Center Research Ethics Committee.

### 2.5 Statistical analysis

Continuous variables are expressed as the means ± standard deviation or medians (interquartile range [IQR]). All categorical variables are presented as the number and percentage in each group. For the whole cohort, comparisons of continuous variables were tested by Student's *t*‐test or Mann–Whitney *U*‐test according to the data distribution. Comparisons of categorical variables were compared by chi‐square analysis or Fisher's exact test. Comparisons of three or more groups were tested by the Kruskal‐Wallis test with post hoc analysis between each group with Bonferroni adjustments.

For assessment of the diagnostic performance of measured parameters, the receiver operating characteristic (ROC) curve was created and the area under the curve (AUC) was calculated.

The estimates of the parameters were given with their 95% confidence intervals (CI). All *P*‐values reported are 2‐sided, and *p* < .05 was considered statistically significant.

All statistical analyses were performed with R (The R Foundation for Statistical Computing, Vienna, Austria, version 3.1.1).

## 3 RESULTS

### 3.1 Patient characteristics

Between September 2017 and June 2020, a total of 93 consecutive patients (52.7% male, age 82.4 ± 9.4 years) undergoing their first Micra leadless pacemaker implantation were included in this analysis. The patient characteristics of the study population are shown in Table 1. The majority of patients were elderly with small body sizes and low BMI. The history of AF was present in 46% of the cases. Cases with AF complicated by complete AV block, which is a good indication for leadless pacemakers, were included in the diagnosis of complete AV block. Even though 46% of patients had a history of heart failure, their LVEF was preserved with a median value of 66.5 (interquartile range [IQR] 58.0–70.3) %.

**TABLE 1:** Baseline characteristics

Number of patients	93
Male, *n* (%)	49 (52.7)
Age, years	82.4 ± 9.4
BMI, kg/m^2^	23.0 ± 4.4
Height, cm	154.4 ± 9.7
Weight, kg	55.4 ± 14.7
Diagnosis	
Complete AVB, *n* (%)	36 (38.7)
Advanced AVB, *n* (%)	9 (9.7)
SSS (I, II), *n* (%)	23 (24.7)
SSS (III), *n* (%)	11 (11.8)
AF with bradycardia, *n* (%)	14 (15.1)
Past medical history	
Hypertension, *n* (%)	45 (48.4)
Diabetes mellitus, *n* (%)	15 (16.1)
Heart failure, *n* (%)	43 (46.2)
AF, *n* (%)	43 (46.2)
IHD, *n* (%)	14 (15.1)
COPD, *n* (%)	7 (7.5)
Hemodialysis, *n* (%)	8 (8.6)
LVEF, %	66.5 (58.0–70.3)

*Note*: Values are reported as the means ± standard deviation, median (interquartile range), or *n* (%).

Abbreviations: AF, atrial fibrillation; AVB, atrioventricular block; BMI, body mass index; COPD, chronic obstructive pulmonary disease; IHD, ischemic heart disease; LVEF, left ventricular ejection fraction; SSS, sick sinus syndrome.

### 3.2 Procedure characteristics

A total of 185 deployments were performed at 93 implantation procedures. The mean and median number of deployments per patient was 2 ± 1.6 and 1 (IQR 1–2), respectively, with a maximum of 8 deployments. The median procedure time from the puncture to sheath removal was 48 (IQR 36–61) minutes. All implantations were successful. The procedural complication of cardiac tamponade occurred in one patient, which required emergent pericardiocentesis.

### 3.3 Visibility of the tine angle from the RAO projection

Since there are 4 tines, a total of 740 tines were assessed by the pull and hold test after 185 deployments. This study only evaluated the RAO projection for reasons previously described. The mean angle of the RAO view was 30.7 ± 5.0 degrees. The poorly visible tines due to overlapping to the body of Micra (*n* = 370 [50%]) and other tines (*n* = 44 [5.9%]) were excluded, leading to 326 (44.1%) visible tines for analysis.

The number of tines evaluated per deployment ranged from 0 to 3 with an average of 1.76 ± 0.66 tines per deployment. Of the 185 deployments, 0, 1, 2, and 3 tines could be assessed in 22 (11.9%), 1 (0.5%), 161 (87.0%), and 1 (0.5%) deployment, respectively.

### 3.4 Differences in tine angles between engaged and not engaged tines

**FIGURE 2:**
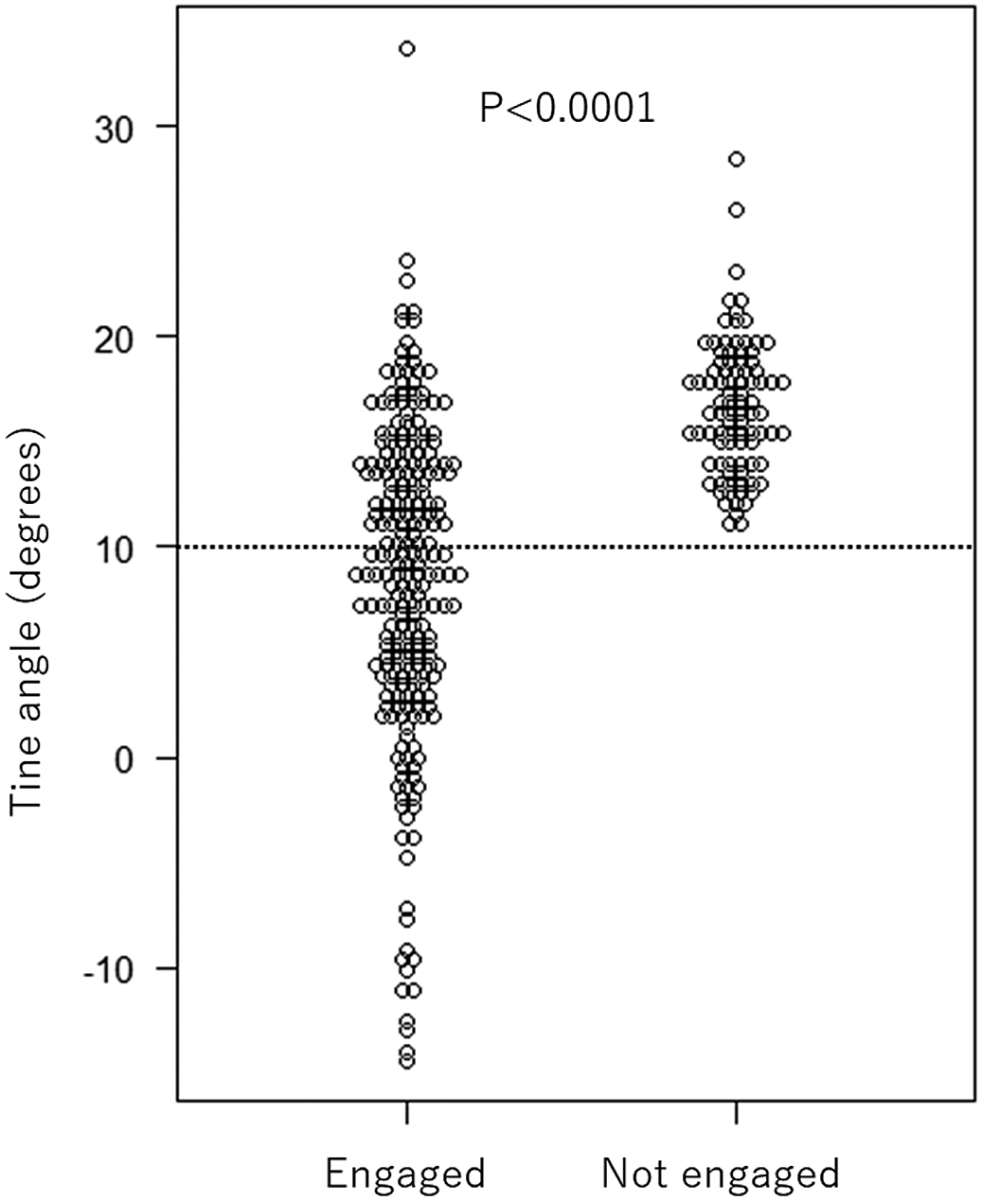
The angle of the tines with and without engagement. The angle of the engaged tines was significantly lower than the non‐engaged tines. All tines with angles <10 degrees were engaged.

**FIGURE 3:**
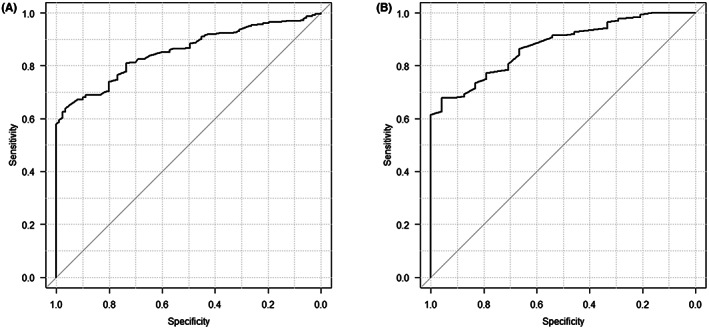
Receiver operating characteristic (ROC) curve for prediction of tine engagement. **(**A) ROC curve of the tine angles for prediction of tine engagement. Area under the curve (AUC) was calculated to be 0.854 (95% CI: 0.815–0.894). (B) ROC curve of the differences in tine angles between contralateral tines for prediction of tine engagement. AUC was calculated to be 0.876 (95% CI: 0.817–0.936).

Of the 326 visible tines, 235 tines were engaged, and 91 tines were not engaged by the pull and hold test. The angle of the engaged tines was significantly lower than the non‐engaged tines (median 9.2 [IQR 4.0–14.0] vs. 16.6 [IQR 14.2–18.8] degrees, *p* < .0001). All tines with angles <10 degrees (*N* = 129) were engaged. In tines with higher angles (*N* = 197), engagement could not be predicted (Figure 2). The cutoff value of less than 10 degrees resulted in positive predictive values of 100%, the negative predictive value of 46.2%, the sensitivity of 54.9%, and the specificity of 100% for the diagnosis of tine engagement. The ROC curve is shown in Figure 3A, which showed the AUC of 0.854 (95% CI: 0.815–0.894).

### 3.5 Differences in tine angles between contralateral tines

**FIGURE 4:**
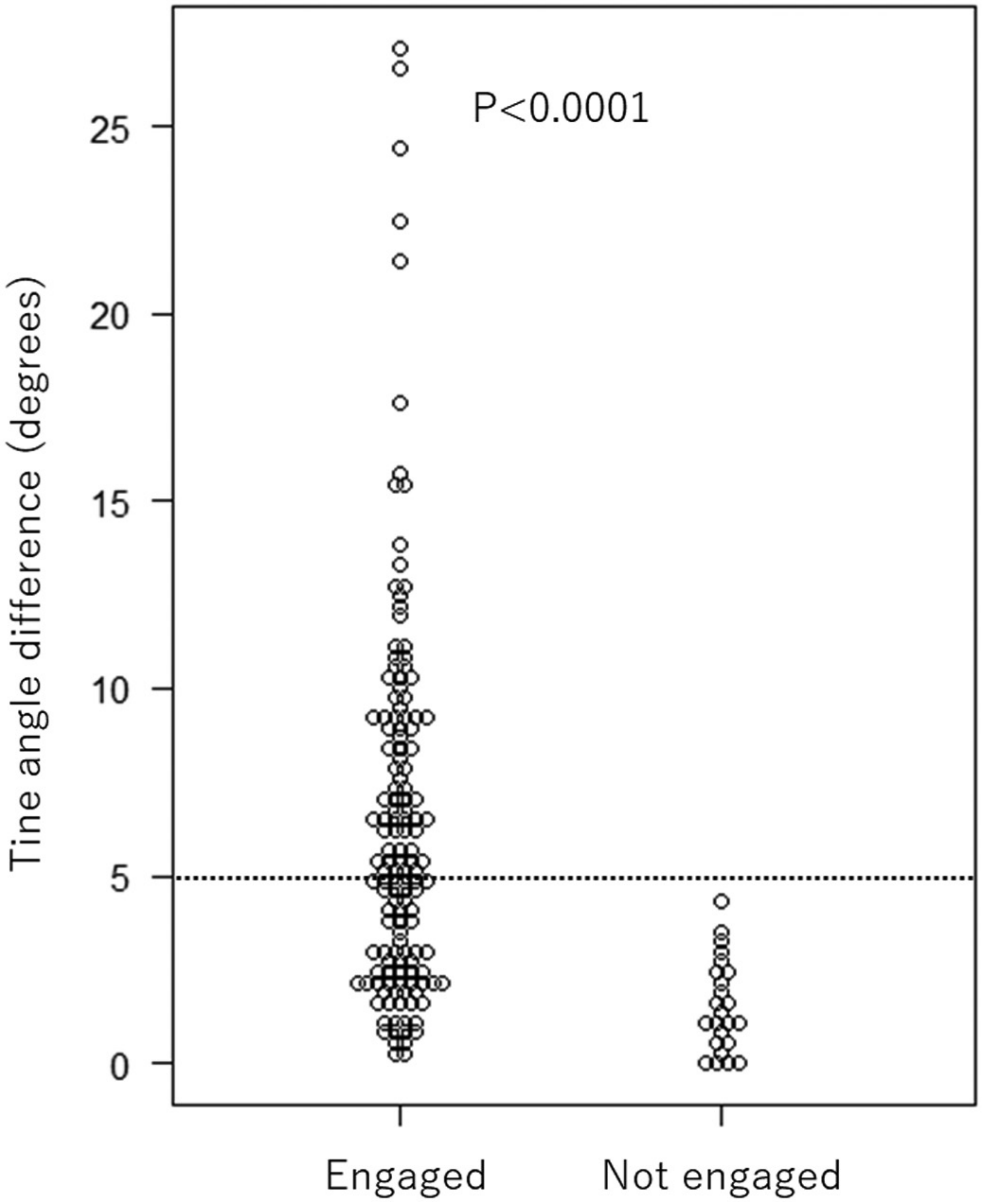
Differences in tine angles between the contralateral tines. The visible tines were paired with contralateral tines. The tines with lower angles compared to the contralateral tines were selected and analyzed for possible engagement. The differences between the engaged tines were significantly higher than the non‐engaged tines. All tines in which the tine angle was >5 degrees lower than the opposing tines were engaged.

Since the tines of Micra are positioned symmetrically, the visible tines were paired with contralateral tines, resulting in 163 pairs. Of the created pairs, the tines with lower angles compared to the opposing tines were selected and analyzed for possible engagement. The differences in the tine angles between each pair were calculated as shown in Figure 1B and were recorded. The angle differences of the engaged tines were significantly higher than the non‐engaged tines (median 5.4 [IQR 2.6–9.0] vs. 1.3 [IQR 0.5–2.4] degrees, *p* < .0001). All tines in which the tine angle was >5 degrees lower than the opposing tines (*N* = 76) were engaged. In lower angle differences (*N* = 87), engagement could not be predicted (Figure 4). The cutoff value of more than 5 degrees resulted in positive predictive values of 100%, the negative predictive value of 27.3%, the sensitivity of 54.3%, and the specificity of 100% for the diagnosis of tine engagement. The ROC curve is shown in Figure 3B, which showed the AUC of 0.876 (95% CI: 0.817–0.936).

**FIGURE 5:**
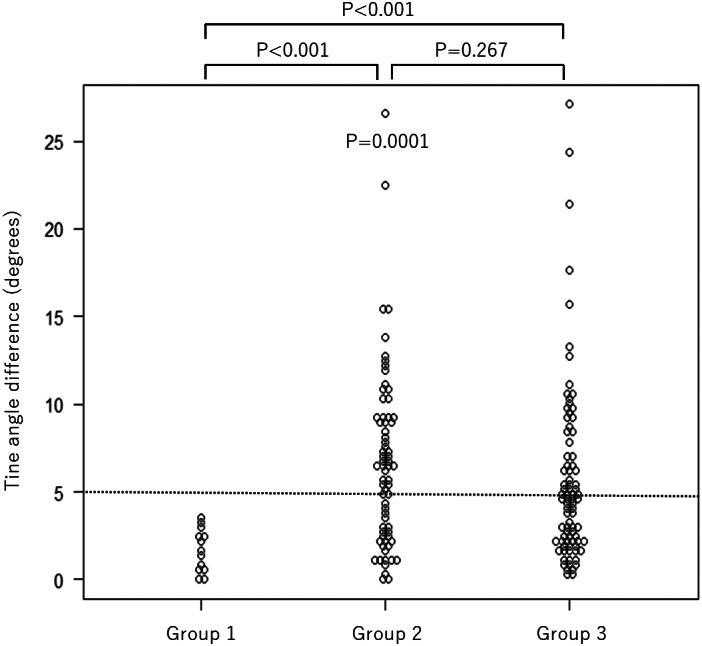
The tine angles between the contralateral tines in different engagement statuses of contralateral tines. The data of difference in tine angle between opposing tines were divided into three groups and compared. Group 1: both sides not engaged. Group 2: only one side engaged. Group 3: both sides engaged. The differences of tine angles between contralateral tines were significantly different between the three groups (*p* = .0001).

Since the engagement status of contralateral tines affects the results, we divided the data into three groups; group 1 with both sides not engaged, group 2 with only one side engaged, and group 3 with both sides engaged (Figure 5). The differences of the tine angles between contralateral tines were significantly different between the three groups (median 1.6 [IQR 0.5–2.4] vs. 6.6 [IQR 2.6–9.1] vs. 4.6 [IQR 2.2–7.5] degrees, respectively, *p* = .0001). In post hoc analysis with Bonferroni adjustment, significant differences were observed between groups 1 and 2 (*p* < .001) and groups 1 and 3 (*p* < .001), but not between groups 2 and 3 (*p* = .267).

### 3.6 Adverse events with the pull and hold test

Of the 326 visible tines, 9 (2.8%) tines were unintendedly dislodged during the pull and hold test, resulting in the need for redeployments. Of those, 7 (77.8%) tines had a tine angle of less than 10 degrees, which is shown to be nearly diagnostic of the tine engagement in this study. Moreover, 3 of the 7 tines also had angles >5 degrees lower than the opposing tines.

### 3.7 Tines in which the engagement could have been predicted before the pull and hold test

**FIGURE 6:**
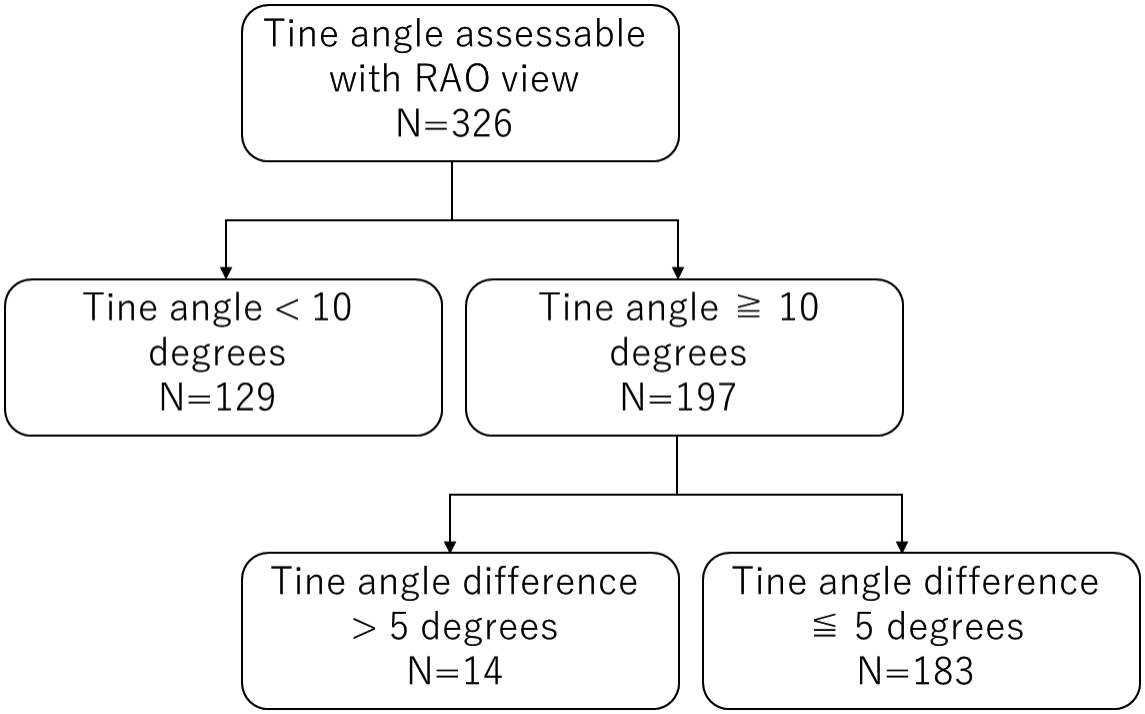
Tines in which the engagement could have been predicted before the pull and hold test. In total, 143 (43.9%) tines could have been predicted as engaged tines before the pull and hold test.

Of the 326 visible tines, 129 (39.6%) tines could have been predicted as engaged tines by tine angle assessment with a cutoff value of <10 degrees. Of the remaining 197 tines, 14 (7.1%) additional tines could have been predicted as engaged by assessment of the difference in tine angle between the contralateral tines with a cutoff value of >5 degrees. In total, 143 (43.9%) tines could have been predicted as engaged tines before the pull and hold test (Figure 6).

The average number of tines that could be diagnosed as engaged by the above methods per deployment was 0.80 ± 0.98.

In 38 out of 185 deployments (20.5%), two or more tines were already open before the pull and hold test which already indicated adequate stability of the device.

## 4 DISCUSSION

The major complications of MICRA leadless pacemaker implantation include cardiac tamponade and device dislodgement.^15^ To minimize the risk of these complications and the number of unnecessary device deployments, assessment of the tine engagement is crucial. However, the pull and hold test was the only method of assessing the engagement.

The tines of Micra are designed to engage in myocardial tissue for the stability of the device. It has been shown in animal models^6^ and in an autopsy of a Micra implanted patient^16^ that the nitinol fixation tines of the device were totally embedded in myocardial tissue. This myocardial tissue entrapped between the tines and the body of Micra may result in a shallower tine angle after deployment if tines are engaged. Our data has clearly shown that the angle of the engaged tines was significantly lower than the non‐engaged tines. To the best of our knowledge, this is the first report which showed that the angle of the tines before the pull and hold test predicts engagement of the tines. Moreover, no other methods of assessing the engagement of the tines other than the pull and hold test have been reported. In the present study, all tines which were already open with a tine angle of less than 10 degrees before the pull and hold test were engaged. These findings may support the assessment of the tine engagements during the procedure.

Pull and hold tests may lead to unintended tine dislodgement, resulting in redeployments. In this study, 9/326 (2.8%) tines were unintendedly dislodged during the pull and hold tests, and many of these tines were already open before the pull and hold test. Since the positive predictive value of the already open tines is 100% in this study, it may be theoretically reasonable to omit the pull and hold test if two or more tines are already open to minimize the risk of unintended tine dislodgements which may lead to complications. However, the safety of omitting the pull and hold test in these populations has not been proven, and tine angle assessment should not be viewed as a guaranteed method of stability assessment.

It has been reported that even though the operator's experience correlates to procedural efficiency, implantation time, and fluoroscopy duration, the number of required deployments has no significant correlation with the operator's experience in leadless pacemaker implantation.^17^ Even the experienced operator requires repeated assessment of the tine engagement, and methods to support the diagnosis are needed.

**FIGURE 7:**
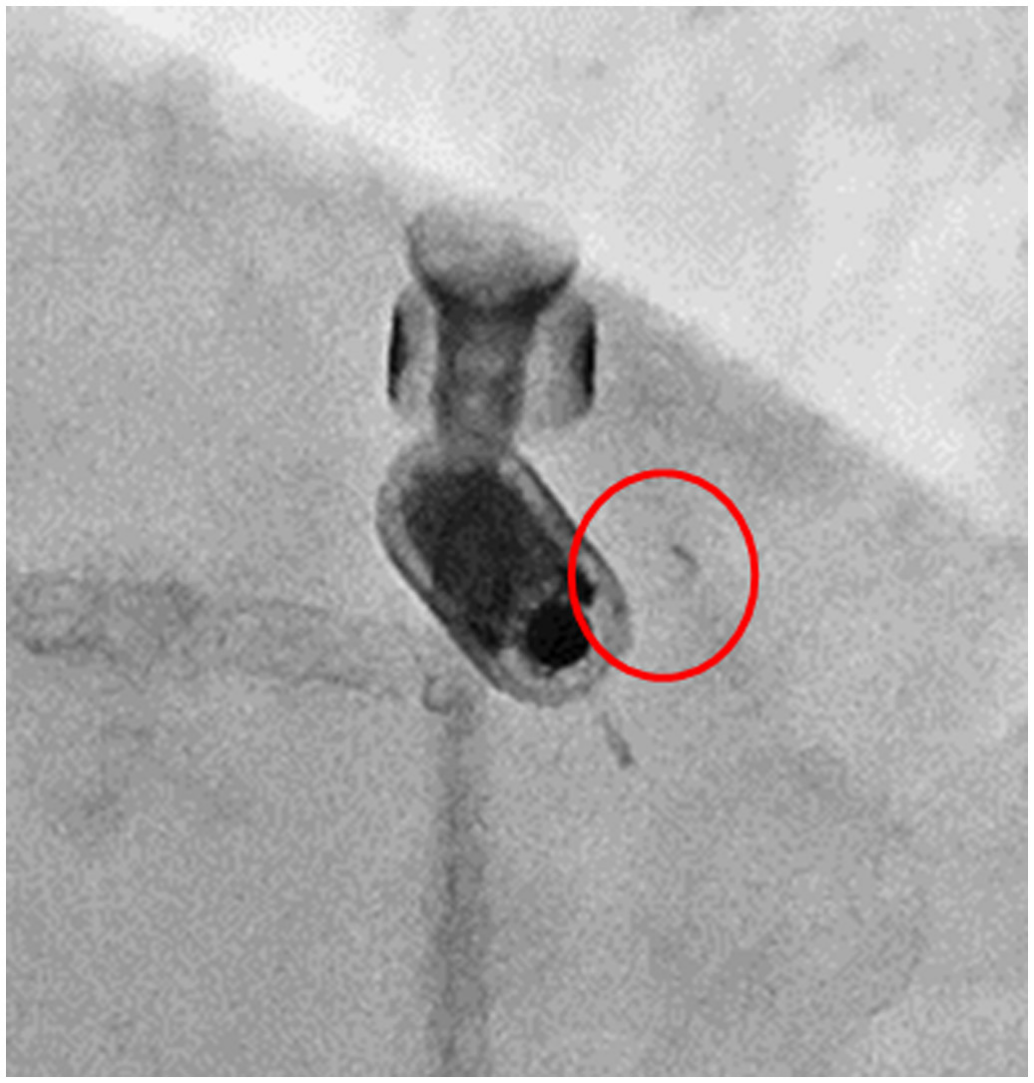
Example of the open tine in LAO view. The septal tine (red circle) is visually open before the pull and hold test with a clear difference between the opposing tine.

LAO view was excluded from the analysis of this study due to difficulty in measuring the angle of the tines. However, it is sometimes obvious that the tine is open before the pull and hold test (Figure 7). From the results of this study, it may be reasonable to believe that these tines are also engaged, even though this study cannot provide data in support of this hypothesis.

### 4.1 Study limitations

This was a retrospective observational single‐center study in Japan, which may have minimal impact on the results of this study.^18^ The angle of the RAO image in relation to the rotation of the heart has not been assessed, which may influence the measured angle of the tines and the results of this study. The cranial and caudal angulation was not analyzed. The strength used by each operator in the pull and hold test are unmeasured and variable, which may have influenced the assessment of the pull and hold test, as well as the rate of tine dislodgement due to the pull and hold test. The operator and the observer who assessed the tine engagement by the pull and hold tests could observe the tines before the pull and hold test, which may have influenced the decision of pull and hold test. Tines that overlap with the body of the leadless pacemaker and other tines were excluded, which may influence the generalizability of the results. Theoretically, our findings may have implications on the assessment of the tines in the LAO view, but data to support this is lacking. The eyeball assessment of the tine angles during the implantation procedure may lack the accuracy to diagnose engagement, especially in border zones. To accurately assess the tine angle, the use of software such as QAngio XA (Medis medical imaging systems, Leiden, Netherlands) may be considered. Theoretically, Group 1 of Figure 5 should have no tine angle differences since both tines are not engaged. However, the measured tine angle differences ranged from 0 to 3.4 degrees, and the presence of measurement error is suggested. Utilization of pull and hold tests should be considered in border zone cases. Even though our findings may support the assessment of the tine engagement, the operator is fully responsible for the final assessment of the tine engagement, as well as the decision to release the device.

## 5 CONCLUSIONS

In conclusion, the low angle of the tines before the pull and hold test can predict engagement of the tines in MICRA leadless pacemaker implantation. The tines which are already open after deployment may be presumed that they are engaged.

## AUTHOR CONTRIBUTIONS

Dr. Mizukami contributed to the conception and design of this work and the drafting of the article. Drs. Shota Miyakuni, Ryo Nakada, Tetsuya Kobayashi, Takuya Kawakami, Koki Takegawa, Hirofumi Arai, Jiro Hiroki, Kenji Yoshioka, Hirofumi Ohtani, Maki Ono, Shu Yamashita, and Daisuke Ueshima contributed to the data acquisition as well as critical revision of the manuscript. Drs. Makoto Suzuki, Akihiko Matsumura, Masahiko Goya, and Tetsuo Sasano contributed to the final approval of the manuscript submitted.

## CONFLICT OF INTEREST

Masahiko Goya received a lecture fee from Johnson and Johnson, Daiichi‐Sankyo, Bayer, and Japan Lifeline and a research grant from Japan Lifeline. The other authors have no conflict of interest to disclose. This research did not receive any specific grant from funding agencies in the public, commercial, or not‐for‐profit sectors.

## ETHICS APPROVAL STATEMENT

This study has been approved by the institutional review board of Kameda Medical Center. (Approval number: 21–067).

## PATIENT CONSENT STATEMENT

In this retrospective observational study, patient consent was obtained by the opt‐out method.

## CLINICAL TRIAL REGISTRATION

Since this is a retrospective study, clinical trial registration was not obtained.

## Data Availability

The data underlying this article will be shared on reasonable request to the corresponding author.

## References

[1] Reynolds D , Duray GZ , Omar R , Soejima K , Neuzil P , Zhang S , et al. A leadless intracardiac transcatheter pacing system. N Engl J Med. 2016;374(6):533–41. 26551877 10.1056/NEJMoa1511643

[2] Ngo L , Nour D , Denman RA , Walters TE , Haqqani HM , Woodman RJ , et al. Safety and efficacy of leadless pacemakers: a systematic review and meta‐analysis. J Am Heart Assoc. 2021;10(13):e019212. 34169736 10.1161/JAHA.120.019212 PMC8403316

[3] Piccini JP , El‐Chami M , Wherry K , Crossley GH , Kowal RC , Stromberg K , et al. Contemporaneous comparison of outcomes among patients implanted with a leadless vs transvenous single‐chamber ventricular pacemaker. JAMA Cardiol. 2021;6(10):1187–95. 34319383 10.1001/jamacardio.2021.2621 PMC8319824

[4] Tjong FV , Reddy VY . Permanent leadless cardiac pacemaker therapy: a comprehensive review. Circulation. 2017;135(15):1458–70. 28396380 10.1161/CIRCULATIONAHA.116.025037

[5] Udo EO , Zuithoff NP , van Hemel NM , de Cock CC , Hendriks T , Doevendans PA , et al. Incidence and predictors of short‐ and long‐term complications in pacemaker therapy: the FOLLOWPACE study. Heart Rhythm. 2012;9(5):728–35. 22182495 10.1016/j.hrthm.2011.12.014

[6] Eggen MD , Grubac V , Bonner MD . Design and evaluation of a novel fixation mechanism for a transcatheter pacemaker. IEEE Trans Biomed Eng. 2015;62(9):2316–23. 26111389 10.1109/TBME.2015.2449320

[7] Ritter P , Duray GZ , Zhang S , Narasimhan C , Soejima K , Omar R , et al. The rationale and design of the Micra transcatheter pacing study: safety and efficacy of a novel miniaturized pacemaker. Europace. 2015;17(5):807–13. 25855677 10.1093/europace/euv026

[8] Ritter P , Duray GZ , Steinwender C , Soejima K , Omar R , Mont L , et al. Early performance of a miniaturized leadless cardiac pacemaker: the Micra Transcatheter Pacing Study. Eur Heart J. 2015;36(37):2510–9. 26045305 10.1093/eurheartj/ehv214 PMC4589655

[9] Mattson AR , Zhingre Sanchez JD , Iaizzo PA . The fixation tines of the Micra leadless pacemaker are atraumatic to the tricuspid valve. Pacing Clin Electrophysiol. 2018;41(12):1606–10. 30341813 10.1111/pace.13529

[10] Hasegawa‐Tamba S , Ikeda Y , Tsutsui K , Kato R , Muramatsu T , Matsumoto K . Two‐directional snare technique to rescue detaching leadless pacemaker. HeartRhythm Case Rep. 2020;6(10):711–4. 33101938 10.1016/j.hrcr.2020.06.027 PMC7573372

[11] Afzal MR , Daoud EG , Cunnane R , Mulpuru SK , Koay A , Hussain A , et al. Techniques for successful early retrieval of the Micra transcatheter pacing system: a worldwide experience. Heart Rhythm. 2018;15(6):841–6. 29427820 10.1016/j.hrthm.2018.02.008

[12] Reddy VY , Miller MA , Knops RE , Neuzil P , Defaye P , Jung W , et al. Retrieval of the leadless cardiac pacemaker: a multicenter experience. Circ Arrhythm Electrophysiol. 2016;9(12):e004626. 27932427 10.1161/CIRCEP.116.004626

[13] JCS Joint Working Group . Guidelines for non‐pharmacotherapy of cardiac arrhythmias (JCS 2011). Circ J. 2013;77(1):249–74. 23165786 10.1253/circj.cj-66-0054

[14] Nogami A , Kurita T , Abe H , Ando K , Ishikawa T , Imai K , et al. JCS/JHRS 2019 guideline on non‐pharmacotherapy of cardiac arrhythmias. J Arrhythm. 2021;37(4):709–870. 34386109 10.1002/joa3.12491 PMC8339126

[15] Roberts PR , Clementy N , Al Samadi F , Garweg C , Martinez‐Sande JL , Iacopino S , et al. A leadless pacemaker in the real‐world setting: the Micra transcatheter pacing system post‐approval registry. Heart Rhythm. 2017;14(9):1375–9. 28502871 10.1016/j.hrthm.2017.05.017

[16] Vamos M , Honold J , Duray GZ , Hohnloser SH . MICRA leadless pacemaker on autopsy. JACC Clin Electrophysiol. 2016;2(5):636–7. 29759585 10.1016/j.jacep.2016.02.014

[17] Haeberlin A , Kozhuharov N , Knecht S , Tanner H , Schaer B , Noti F , et al. Leadless pacemaker implantation quality: importance of the operator's experience. Europace. 2020;22(6):939–46. 32361742 10.1093/europace/euaa097

[18] Soejima K , Asano T , Ishikawa T , Kusano K , Sato T , Okamura H , et al. Performance of leadless pacemaker in Japanese patients vs. rest of the world‐ results from a global clinical trial. Circ J. 2017;81(11):1589–95. 28566657 10.1253/circj.CJ-17-0259

